# Gene-expression signature regulated by the KEAP1-NRF2-CUL3 axis is associated with a poor prognosis in head and neck squamous cell cancer

**DOI:** 10.1186/s12885-017-3907-z

**Published:** 2018-01-06

**Authors:** Akhileshwar Namani, Md. Matiur Rahaman, Ming Chen, Xiuwen Tang

**Affiliations:** 1 Department of Biochemistry, University School of Medicine, Hangzhou, 310058 People’s Republic of China; 2 Department of Bioinformatics, College of Life Sciences, Zhejiang University, Hangzhou, 310058 People’s Republic of China

**Keywords:** Head and neck squamous cell cancer, KEAP1-NRF2-CUL3 mutations, Overall survival, Gene-expression signature

## Abstract

**Background:**

NRF2 is the key regulator of oxidative stress in normal cells and aberrant expression of the NRF2 pathway due to genetic alterations in the KEAP1 (Kelch-like ECH-associated protein 1)-NRF2 (nuclear factor erythroid 2 like 2)-CUL3 (cullin 3) axis leads to tumorigenesis and drug resistance in many cancers including head and neck squamous cell cancer (HNSCC). The main goal of this study was to identify specific genes regulated by the KEAP1-NRF2-CUL3 axis in HNSCC patients, to assess the prognostic value of this gene signature in different cohorts, and to reveal potential biomarkers.

**Methods:**

RNA-Seq V2 level 3 data from 279 tumor samples along with 37 adjacent normal samples from patients enrolled in the The Cancer Genome Atlas (TCGA)-HNSCC study were used to identify upregulated genes using two methods (altered KEAP1-NRF2-CUL3 versus normal, and altered KEAP1-NRF2-CUL3 versus wild-type). We then used a new approach to identify the combined gene signature by integrating both datasets and subsequently tested this signature in 4 independent HNSCC datasets to assess its prognostic value. In addition, functional annotation using the DAVID v6.8 database and protein-protein interaction (PPI) analysis using the STRING v10 database were performed on the signature.

**Results:**

A signature composed of a subset of 17 genes regulated by the KEAP1-NRF2-CUL3 axis was identified by overlapping both the upregulated genes of altered versus normal (251 genes) and altered versus wild-type (25 genes) datasets. We showed that increased expression was significantly associated with poor survival in 4 independent HNSCC datasets, including the TCGA-HNSCC dataset. Furthermore, Gene Ontology, Kyoto Encyclopedia of Genes and Genomes, and PPI analysis revealed that most of the genes in this signature are associated with drug metabolism and glutathione metabolic pathways.

**Conclusions:**

Altogether, our study emphasizes the discovery of a gene signature regulated by the KEAP1-NRF2-CUL3 axis which is strongly associated with tumorigenesis and drug resistance in HNSCC. This 17-gene signature provides potential biomarkers and therapeutic targets for HNSCC cases in which the NRF2 pathway is activated.

**Electronic supplementary material:**

The online version of this article (10.1186/s12885-017-3907-z) contains supplementary material, which is available to authorized users.

## Background

Head and neck squamous cell cancer (HNSCC) is the sixth most prevalent form of cancer. It has a high incidence worldwide, and 90% of cases are histologically identified as squamous cell carcinomas [1, 2]. HNSCC is a broad category of cancers that predominantly arise in the oral cavity, oropharynx, hypopharynx, larynx, soft tissues of the neck, salivary glands, skin, and mucosal membranes [3, 4]. The most common causes are the consumption of tobacco and alcohol, and human papillomavirus infection [5].

NRF2 is the master transcription factor that regulates the genes involved in antioxidant and detoxification pathways. Under normal conditions, Kelch like-ECH-associated protein 1 (KEAP1) negatively regulates the NRF2 expression by cullin-3 (CUL3)-mediated ubiquitination and proteasomal degradation [6]. Under oxidative stress, NRF2 is liberated from the tight control of the KEAP1/CUL3 complex, is relocated to the nucleus where it forms heterodimers with small Maf proteins, and transactivates its downstream genes through binding with antioxidant responsive elements (AREs) [7]. Genetic alterations such as mutations (gain of function mutations of NRF2 and loss of function mutations in KEAP1 and CUL3), and copy-number changes (amplification of NRF2 and deletion of KEAP1 and CUL3) leads to oncogenesis and drug- and radio-resistance in different types of cancers including HNSCC [8, 9]. Due to the dysregulated NRF2 activity in different cancers, it is emerging as a promising therapeutic target in drug discovery [10, 11].

Stacy et al. [12] first reported the increased expression of NRF2 in HNSCC patients and suggested that NRF2 might be a biomarker. Another report from Huang et al. [13] found the increased expression of KEAP1 and NRF2 in oral squamous cell carcinoma. However, in their report, overall survival analysis of patients with increased expression of KEAP1 and NRF2 did not reveal significant differences. Recently, The Cancer Genome Atlas (TCGA) has provided a wealth of information about KEAP1-NRF2-CUL3 changes in HNSCC patients [14]. Therefore, examining the molecular mechanisms involved in these alterations by using publicly available data may contribute to the development and design of therapeutic targets for personalized/precision medicine in subsets of patients. Several emerging studies including our recent study on lung cancer have identified an NRF2-regulated gene signature and potential biomarkers for patient survival and NRF2 activity [15–18].

Given the importance of KEAP1-NRF2-CUL3 changes in HNSCC, it is important to identify the biomarkers that determine patient survival and NRF2 activity. A recent analysis on TCGA-HNSCC data revealed that patients with disruption of the KEAP1/CUL3/RBX1 E3-ubiquitin ligase complex have significantly poorer survival than non-disrupted counterparts [19]. However, their study specifically focused on the data from patients with a disrupted KEAP1/CUL3/RBX1 complex, but not the data from samples in which NRF2 was altered. In addition they utilized 302 patients data which contains provisional information in their study and overall survival analysis was limited to one cohort. In our study, we restricted the patients samples number (*n* = 279) which were reported in the TCGA publication [14] and excluded provisional data. Moreover, we analyzed the TCGA-HNSCC [14] RNA-Seq data and identified a 17-gene signature that was highly expressed in samples with altered KEAP1-NRF2-CUL3 compared with both normal and wild-type counterparts. Further, we showed that genomic changes in KEAP1-NRF2-CUL3 were key effectors of the overexpression of genes dependent on the NRF2 pathway. Furthermore, we identified known NRF2-regulated genes involved in drug and glutathione metabolism, along with 4 putative KEAP1-NRF2-CUL3-regulated genes. Finally, we found that higher expression of this gene signature was significantly associated with poorer survival in 4 HNSCC cohorts.

## Methods

### Samples and transcriptomic profile datasets

We obtained RNA-Seq gene expression version2 (RNA-SeqV2) level 3 data (Illumina Hiseq platform) from HNSCC patients along with adjacent normal tissues from the Broad GDAC Firehose website (http://gdac.broadinstitute.org/). We carried out the analysis of RNA-Seq data of 279 tumor samples and 37 adjacent normal samples listed in the TCGA network study [14]. All the alteration data for KEAP1-NRF2-CUL3 (KEAP1-mutation/deletion, NRF2-mutation/amplification, and CUL3-muatation/deletion) used in the present study was obtained from cBioportal [20, 21]. In addition to the TCGA-HNSCC RNA-Seq data, three independent HNSCC cohorts microarray data– Saintigny et al. (GSE26549) [22], Jung et al. (E-MTAB-1328) [23], and Cohen et al. (GSE10300) [24] – were also used for overall survival analysis. Our study meets the publication guidelines listed by the TCGA network.

### RNA-Seq data analysis

The conventional method of differentially-expressed gene (DEG) analysis involves the comparison of tumor transcriptomic data with normal cell data. However, in recent studies, due to the availability of large sets of tumor samples and fewer adjacent normal datasets, researchers have performed DEG analysis of TCGA data by applying a new method in which the DEGs are identified by comparing altered or mutated tumor samples (including a particular gene/set of genes) with wild-type tumors (caused by factors other than alterations or mutations) [15, 25, 26].

Despite the fact that these two methods have been used separately for DEG analysis, in this study, we applied a combinatorial approach to obtain DEGs from HNSCC patients by using both conventional and new methods. We then integrated the resulting upregulated genes from both datasets to obtain overlapping genes. This approach led to the robust identification of more markedly upregulated genes specific to the samples with altered KEAP1-NRF2-CUL3 than in both normal and wild-type samples. Moreover, our method not only identified specific genes targeted by the KEAP1-NRF2-CUL3 axis but also minimized false-positive results.

**Fig. 1:**
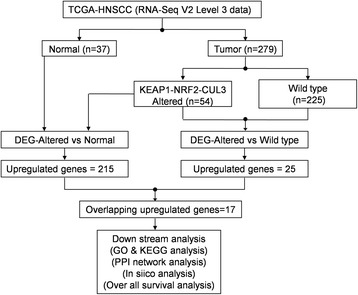
Overview of transcriptomic analysis of TCGA-HNSCC RNA-Seq data. DEG, differentially-expressed genes

We segregated the 279 HNSCC tumor samples into two groups: 54 altered KEAP1-NRF2-CUL3 samples (referred to below as ‘altered’) and 225 wild-type samples. Before performing transcriptomic data analysis, the TCGA barcodes of patient data were cross-checked to avoid technical errors. First, we carried out DEG analysis in the 54 altered versus 37 normal samples followed by 54 altered versus 225 wild-type samples using the R/Bioconductor package [27] – edgeR [28]. To crosscheck how our combinatorial approach effectively found specific genes targeted by the KEAP1-NRF2-CUL3 axis, we also subjected the 225 wild-type and 37 normal samples to DEG analysis. Briefly, the raw counts of RNA-SeqV2 level 3 data were filtered by removing the genes containing zero values. We then considered the genes with >100 counts per million in at least two samples for normalization using the trimmed mean of M-values method, followed by the estimation of dispersions using generalized linear models. Up- and down-regulated genes for altered versus normal and altered versus wild-type samples were identified separately by applying a Benjamini-Hochberg (BH) false-discovery rate (FDR) *p* < 0.01 with a log-fold change (logFC) > 1.5 and <−1.5. Finally, we used the overlapping upregulated genes obtained from both datasets using ‘Venny 2.1’ (http://bioinfogp.cnb.csic.es/tools/venny/index.html) for further analysis. Hierarchical clustering of overlapping upregulated genes was performed using the ‘Heatmapper’ web tool [29]. Box plots of the overlapping upregulated genes that represent the log (counts per million) expression values were generated using R-package ‘ggplot2’ [30]. The overall workflow of the study design is presented in Fig. 1.

### Functional annotation and protein-protein interaction (PPI) network analysis

Functional annotation (Gene Ontology (GO) and Kyoto Encyclopedia of Genes and Genomes (KEGG) analysis) of overlapping upregulated genes was performed using the updated version of the Database for Annotation, Visualization and Integrated Discovery (DAVID) v6.8 web tool [31]. PPI network analysis was performed using the STRING v10 database [32].

### Identification of NRF2-binding sites by in silico analysis

To identify the NRF2 binding sites within the promoter regions of the putative KEAP1-NRF2-CUL3-regulated genes, we used the transcription factor-binding site finding tool LASAGNA-Search 2.0 [33] with cutoff *p*-values ≤ 0.001. The search was limited to the -5 kb upstream promoter region relative to the transcription start site.

### Survival analysis

Cox proportional hazard regression was performed using the online survival analysis and biomarker validation tool SurvExpress [34]. We considered the data from a total of 502 patients in 4 independent HNSCC cohorts available in the SurvExpress database: the TCGA-HNSCC cohort (*n* = 283) with other three HNSCC cohorts – Saintigny et al. (GSE26549) (*n* = 86) [22], Jung et al. (E-MTAB-1328) (*n* = 89) [23], and Cohen et al. (GSE10300) (*n* = 44) [24] – for survival analysis. In the case of microarray-based survival data, we considered the average values for genes whose expression was associated with multiple probe sets such as duplicates or alternatives. SurvExpress separated the patient samples into two groups, high - and low-risk, based on average expression of the 17 genes signature values, and performed statistical analysis of survival probability of the two groups using the log-rank method. SurvExpress used the log-rank test to generate Kaplan-Meir plots based on the ‘Survival’ package of the R platform, which is integrated into its website. Log-rank test *p*-values < 0.05 were considered to be statistically significant.

## Results

### Overview of genetic alterations in the KEAP1-NRF2-CUL3axis

**Fig. 2:**
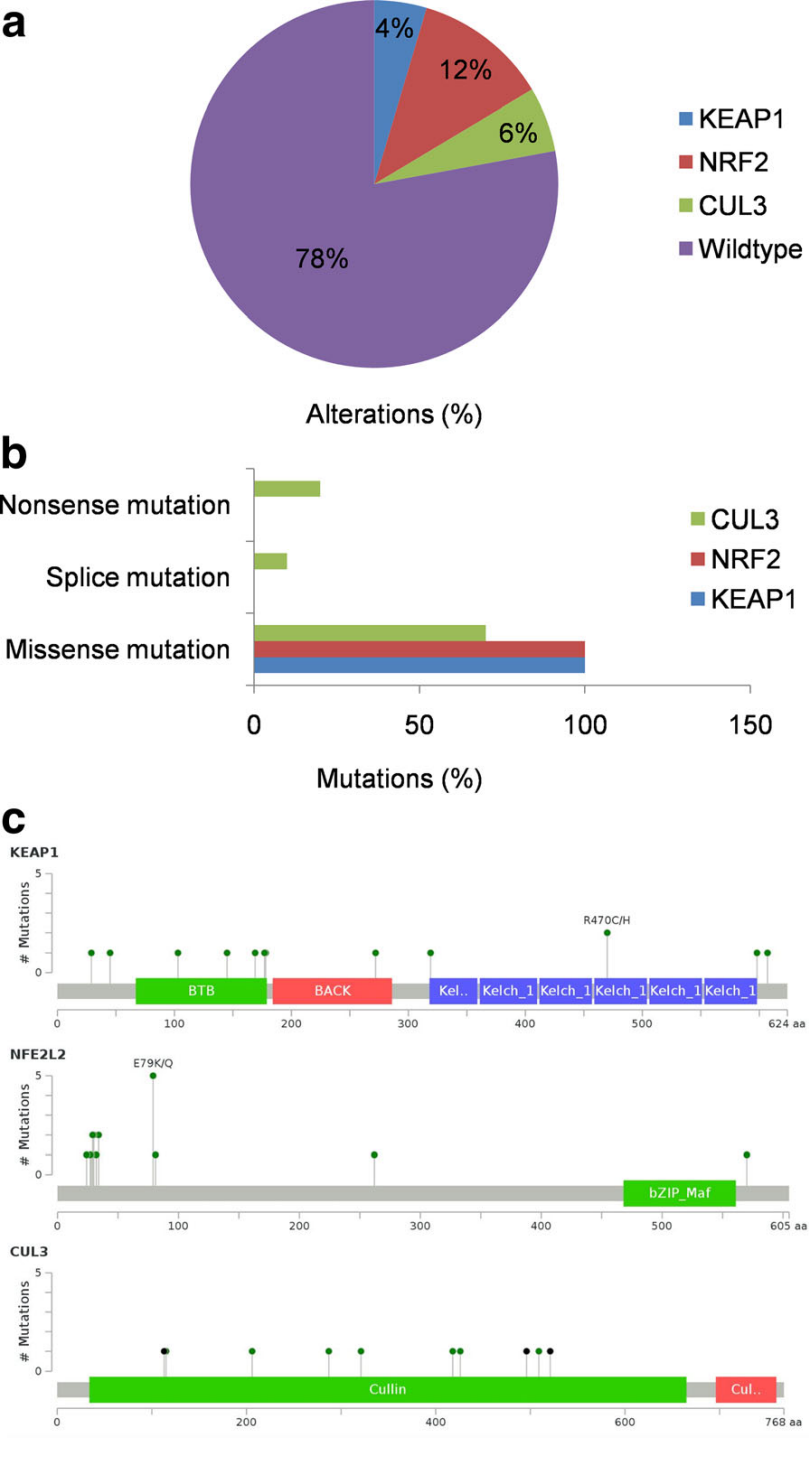
Overview of genetic changes in KEAP1-NRF2-CUL3 in TCGA-HNSCC patients. **a** Pie chart showing individual percentages of genetic alterations in the KEAP1-NRF2-CUL3 complex. **b** Bar chart showing the types and percentages of mutations of the KEAP1-NRF2-CUL3 complex. **c** cBioportal-predicted mutation maps (lollipop plots) showing the positions of mutations on the functional domains of KEAP1, NRF2, and CUL3 proteins. The colored lollipops show the positions of the mutations as identified by whole-exon sequencing

In HNSCC, changes in the KEAP1-NRF2-CUL3 axis occurred in ~20% of patients; of these, KEAP1 alterations accounted for 4.6%, NRF2 for 11.8%, and CUL3 for 5.7%. However, few samples overlapped (Fig. 2a). In order to better understand the KEAP1-NRF2-CUL3 mutational landscape in HNSCC, we used the cBioportal cancer genomics website [20, 21] to examine the types of mutation and their positions in the domain structure of proteins. All 13 KEAP1 and 18 NRF2 mutations were missense mutations, while 70% of the CUL3 mutations (7/10) were missense, 20% (2/10) were nonsense, and 10% (1/10) were splice mutations (Fig. 2b).

KEAP1 consists of 605 amino-acids with 3 domains in which 6 mutations were reported in the BTB (broad-complex, tramtrack, and bric-a-brac) domain, 1 in the IVR (intervening region), 1 in the C-terminal, 1 in the N-terminal region, and another 4 were in the Kelch domain, which is essential for the binding of NRF2. In the case of NRF2 structure, the majority of mutations (16) occurred in the crucial KEAP1-binding domain Neh2, and another 2 were found in each of the Neh7 and Neh3 domains. CUL3 contained 4 mutations in the N-terminal domain, 5 in the C-terminal domain, and 1 in the cullin repeat 3 domain (Fig. 2c). Overall, two samples contained both KEAP1 and NRF2 mutations, while one sample contained both NRF2 and CUL3 mutations. KEAP1 and CUL3 mutations were mutually exclusive.

### Identification of genes regulated by the KEAP1-NRF2-CUL3 axis in HNSCC

**Fig. 3:**
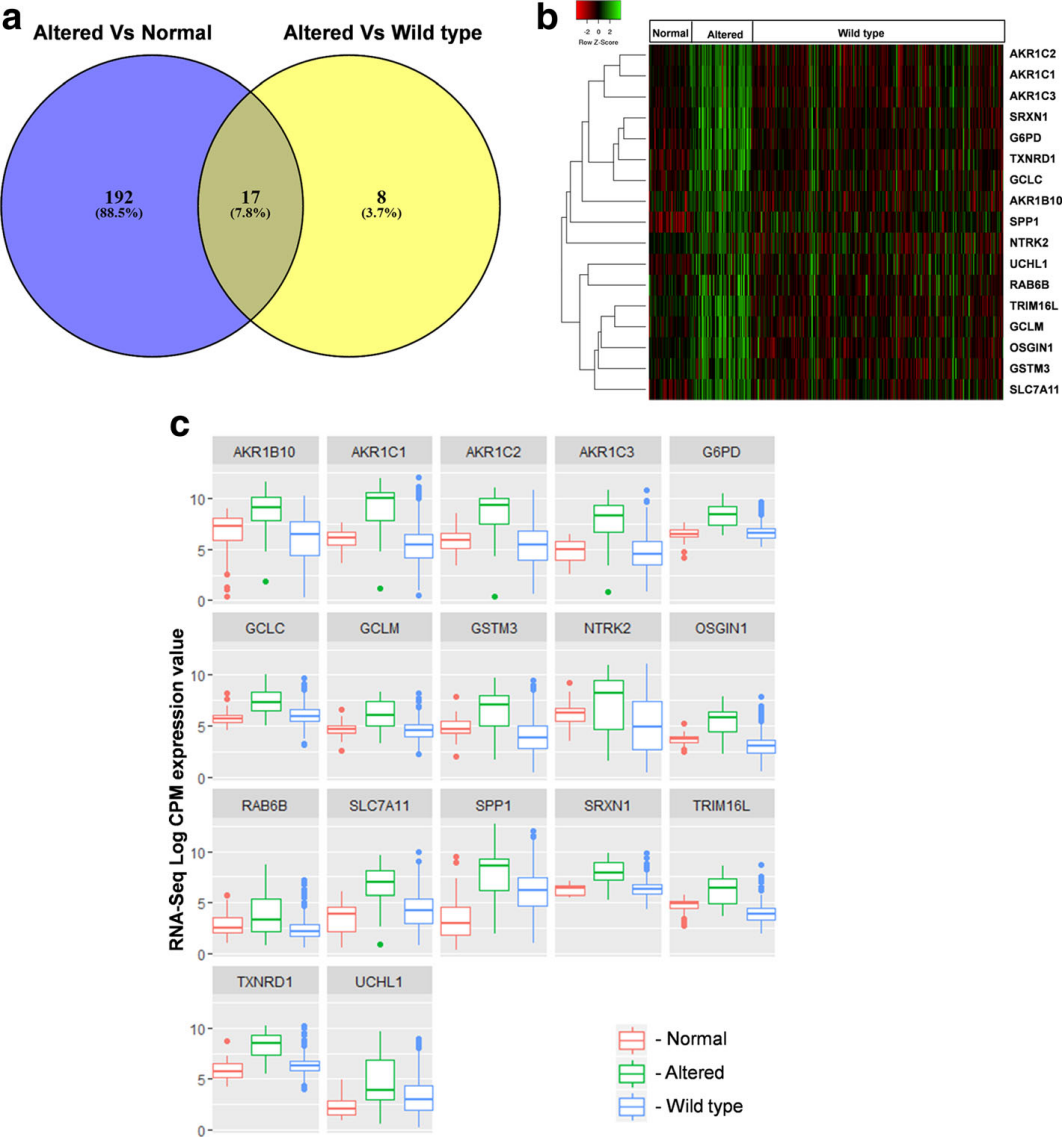
Identification of expression signature of genes regulated by KEAP1-NRF2-CUL3 axis in TCGA-HNSCC. **a** Venn diagram of overlapping genes from both altered versus normal and altered versus wild-type upregulated gene analysis in HNSCC. **b** Hierarchical clustering of normal, altered, and wild-type cases showing the specific expression pattern of the 17-gene signature. Green, relatively high expression; red, relatively low expression. **c** Box plots of 17-gene signature illustrating significant differences of expression in normal, altered, and wild-type cases. X-axis, RNA-Seq V2 log CPM (counts per million) values

In order to identify the genes regulated by the KEAP1-NRF2-CUL3 axis in HNSCC, we focused on the identification of differentially expressed genes by analyzing the RNA-Seq expression profiles in 54 altered versus 37 normal, and 54 altered versus 225 wild-type samples. A total of 215 upregulated genes and 9 downregulated genes were found in the altered versus normal analysis (Additional file 1: Table S1), and 25 upregulated genes and 13 downregulated genes in the altered versus wild-type analysis (Additional file 2: Table S2) with logFC >1.5 (*p* < 0.01 with BH-FDR adjustment). Since the ultimate effect of KEAP1-NRF2-CUL3 axis gene alterations leads to overexpression of NRF2 and its downstream genes, we focused on the upregulated genes for further analysis. By integrating both datasets using Venny web tool (http://bioinfogp.cnb.csic.es/tools/venny/index.html), we obtained 17 overlapping upregulated genes (Fig. 3a). We carried out literature survey to verify whether the downregulated genes obtained from both methods contains previously reported NRF2 regulated genes or not. Notably, we didn’t observe any previously reported NRF2 target genes among all downregulated genes.

We also carried out DEG analysis in 225 wild-type versus 37 normal samples to assess the specificity of the 17 genes regulated by the KEAP1-NRF2-CUL3 axis. Strikingly, none of the 17 genes were found in the list of upregulated genes in the wild-type versus normal samples with logFC > 1.5 (*p* < 0.01 with BH-FDR adjustment; Additional file 3: Table S3). Thus, our analysis clearly showed that these 17 genes were significantly overexpressed in altered KEAP1-NRF2-CUL3 samples compared with their normal and wild-type counterparts (Fig. 3b, c). We then designated these 17 genes as the signature of gene expression regulated by the KEAP1-NRF2-CUL3 axis based on their specificity and higher expression (Table 1). Among these 17 genes, 13 – AKR1B10, AKR1C1, AKR1C2, AKR1C3, G6PD, GCLC, GCLM, GSTM3, OSGIN1, SRXN1, TXNRD1, SLC7A11 [11, 35, 36], and SPP1 [37]– are well-known NRF2-regulated genes, listed and reviewed in a wide variety of studies.

**Table 1:** List of 17 upregulated KEAP1-NRF2-CUL3 axis genes identified in HNSCC

Gene symbol	Description
AKR1B10	Aldo-keto reductase family 1 member B10
AKR1C1	Aldo-keto reductase family 1 member C1
AKR1C2	Aldo-keto reductase family 1 member C2
AKR1C3	Aldo-keto reductase family 1 member C3
G6PD	Glucose-6-phosphate dehydrogenase
GCLC	Glutamate-cysteine ligase catalytic subunit
GCLM	Glutamate-cysteine ligase modifier subunit
GSTM3	Glutathione S-transferase mu 3
NTRK2	Neurotrophic receptor tyrosine kinase 2
OSGIN1	Oxidative stress induced growth inhibitor 1
RAB6B	RAB6B, member RAS oncogene family
SLC7A11	Solute carrier family 7 member 11
SPP1	Secreted phosphoprotein 1
SRXN1	Sulfiredoxin 1
TRIM16L	Tripartite motif containing 16-like
TXNRD1	Thioredoxin reductase 1
UCHL1	Ubiquitin C-terminal hydrolase L1

### NRF2 binds with the ARE sequences of 3 putative genes identified in the 17-gene signature

**Fig. 4:**
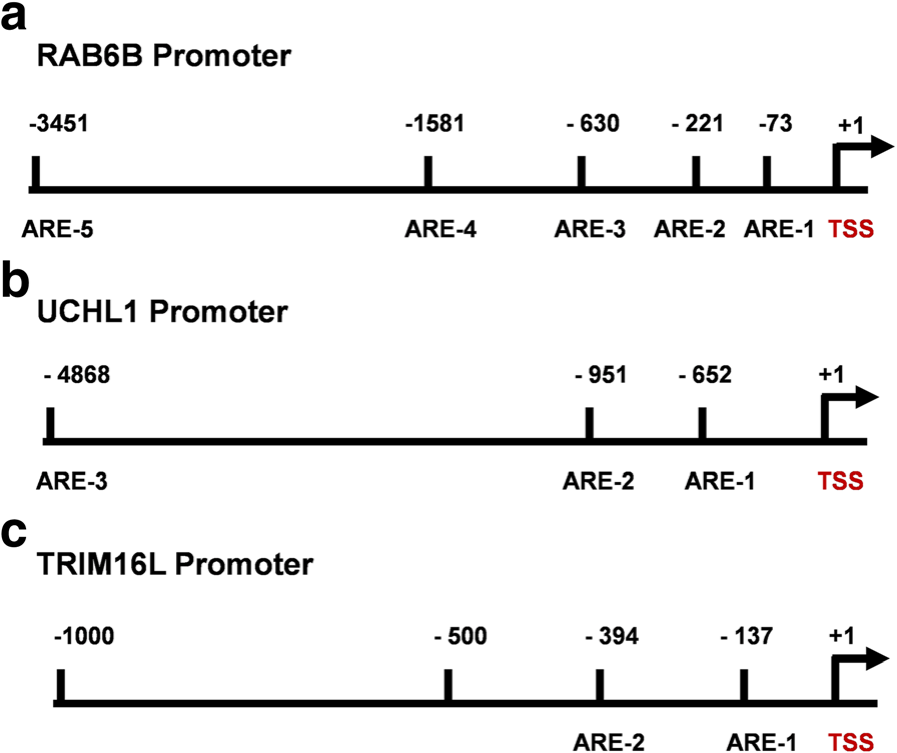
In silico analysis of NRF2 binding sites. Schematic representation shows positions of in silico predicted NRF2 binding sites (AREs) in the promoter regions of human (**a**), RAB6B, (**b**), UCHL1, (**c**), TRIM16L genes

Since the ultimate effect of KEAP1-NRF2-CUL3 gene alterations results in the overexpression of NRF2 and its target genes, it was not surprising that the majority of genes in our results were well-characterized NRF2-regulated genes. In addition, we found 4 putative KEAP1-NRF2-CUL3-regulated genes, NTRK2 (neurotrophic receptor tyrosine kinase 2), RAB6B, TRIM16L, and UCHL1 and investigated whether they were also regulated by NRF2. Interestingly, further in silico analysis using the ‘LASAGNA-Search 2.0’ [33] bioinformatics tool identified NRF2-ARE sequences within the -5 kb upstream promoter regions of the human RAB6B, UCHL1 and TRIM16L genes (Fig. 4a,b,c; Additional file 4: Table S4). However, we did not find an ARE sequence in the promoter region of the NTRK2 gene. Together, our results suggest that NRF2 directly binds with the promoter regions of 16 of the genes in the signature and triggers their overexpression; NTRK2 is the exception.

### Functional annotation of the gene expression signature regulated by the KEAP1-NRF2-CUL3 axis

Functional annotation analysis from GO and KEGG pathway predictions using both DAVID and STRING v10 revealed that the 17 genes were significantly enriched (*p* < 0.001) in the biological processes daunorubicin metabolic process, doxorubicin metabolic process, oxidation-reduction process, cellular response to jasmonic acid stimulus, progesterone metabolic process, response to oxidative stress, and steroid metabolic process. In KEGG pathway analysis, we found significant enrichment (*p* < 0.005) in the three pathways glutathione metabolism, steroid hormone biosynthesis, and metabolism of xenobiotics by cytochrome P450 (Table 2).

**Table 2.** GO and KEGG pathway analysis of 17 KEAP1-NRF2-CUL3 axis regulated genes in HNSCC

Term	*p*-value	Genes
GO_Biological Proceess (GO_BP)		
GO:0044597~daunorubicin metabolic process	3.22E-08	AKR1C3, AKR1C2, AKR1B10, AKR1C1
GO:0044598~doxorubicin metabolic process	3.22E-08	AKR1C3, AKR1C2, AKR1B10, AKR1C1
GO:0055114~oxidation-reduction process	3.29E-07	AKR1C3, AKR1C2, G6PD, AKR1B10, OSGIN1, TXNRD1, AKR1C1, SRXN1
GO:0071395~cellular response to jasmonic acid stimulus	4.46E-06	AKR1C3, AKR1C2, AKR1C1
GO:0042448~progesterone metabolic process	2.67E-05	AKR1C3, AKR1C2, AKR1C1
GO:0006979~response to oxidative stress	1.18E-04	GCLC, GCLM, SRXN1, SLC7A11
GO:0008202~steroid metabolic process	6.58E-04	AKR1C3, AKR1C2, AKR1B10
KEGG Pathway		
hsa00480:Glutathione metabolism	5.9956E-05	GSTM3, G6PD, GCLC, GCLM
hsa00140:Steroid hormone biosynthesis	0.00362777	AKR1C3, AKR1C2, AKR1C1
hsa00980:Metabolism of xenobiotics by cytochrome P450	0.00584594	AKR1C2, GSTM3, AKR1C1

### The 17-gene signature is significantly associated with poor survival in TCGA-HNSCC patients

**Fig. 5:**
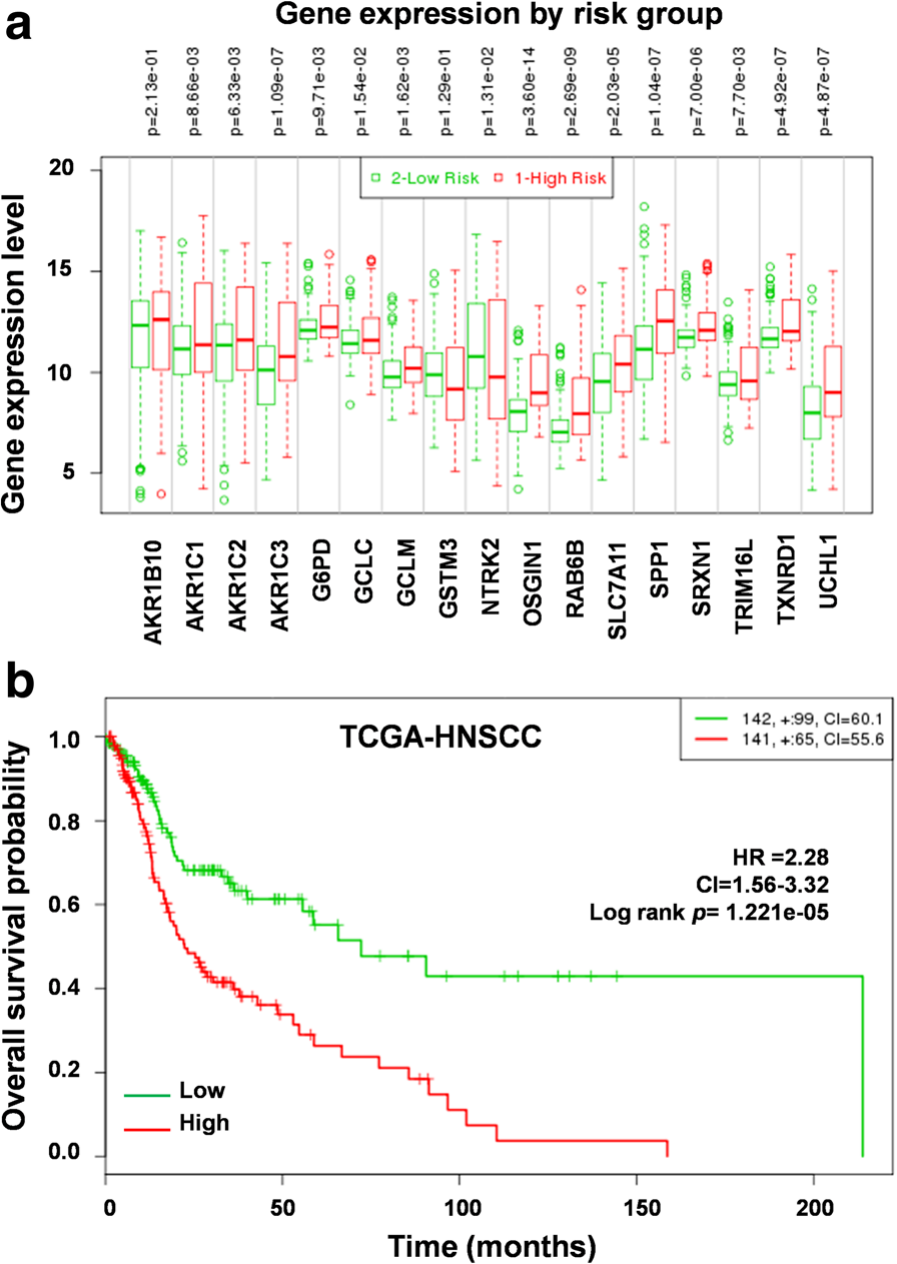
Correlation of 17-gene signature with poor survival in TCGA-HNSCC patients. **a** Box plots of the expression differences of the 17-gene signature in low (green) and high (red) risk groups of TCGA-HNSCC patients. X-axis, gene expression value of each gene; above the box plot, *p*-values of the expression difference between risk groups. **b** Kaplan-Meier survival plots showing that high expression of the 17-gene signature is associated with poor survival in TCGA-HNSCC patients. Red, high-risk group; green, low-risk group; top right corner inset, numbers of high- and low-risk samples, numbers of censored samples marked with + and concordance index (CI) of each risk group; X-axis, time (months); Y-axis, overall survival probability; HR, hazard ratio; CI, confidence interval

To evaluate the prognostic value of the 17-gene signature in patient survival, we first analyzed overall survival in the TCGA-HNSCC cohort available in the SurvExpress web tool. A total of 283 patient samples were divided into high-risk (*n* = 141) and low-risk groups (*n* = 142) based on their expression pattern (Fig. 5a). The survival probability estimates in the two risk groups were visualized as Kaplan-Meier plots. Strikingly, overall survival analysis revealed that the patients in the high-risk group had poorer survival (HR = 2.28; CI = 1.56–3.32; *p* = 1.221e-05) than the low-risk group (Fig. 5b). Thus, our analysis strongly suggests that genes regulated by the KEAP1-NRF2-CUL3 axis are powerful predictors of a poor prognosis in HNSCC patients. In addition, we also carried out the multivariate analysis with the limited variables present in Survexpress database. Consistent with the above results, patients with high-risk scores for clinical variables such as tumor grades G2 and G3, pathological stages T1 and T2, and pathological disease stages II and III were significantly associated with poor survival whereas the results were insignificant in other variables (Additional file 5: Table S5). Kaplan-Meier survival plots with log-rank test results for the significant clinical variables are shown in Additional file 6: Figure S1.

### Association of 17-gene signature with disease-free survival (DFS), metastasis-free survival (MFS), and recurrence in HNSCC patients

**Fig. 6:**
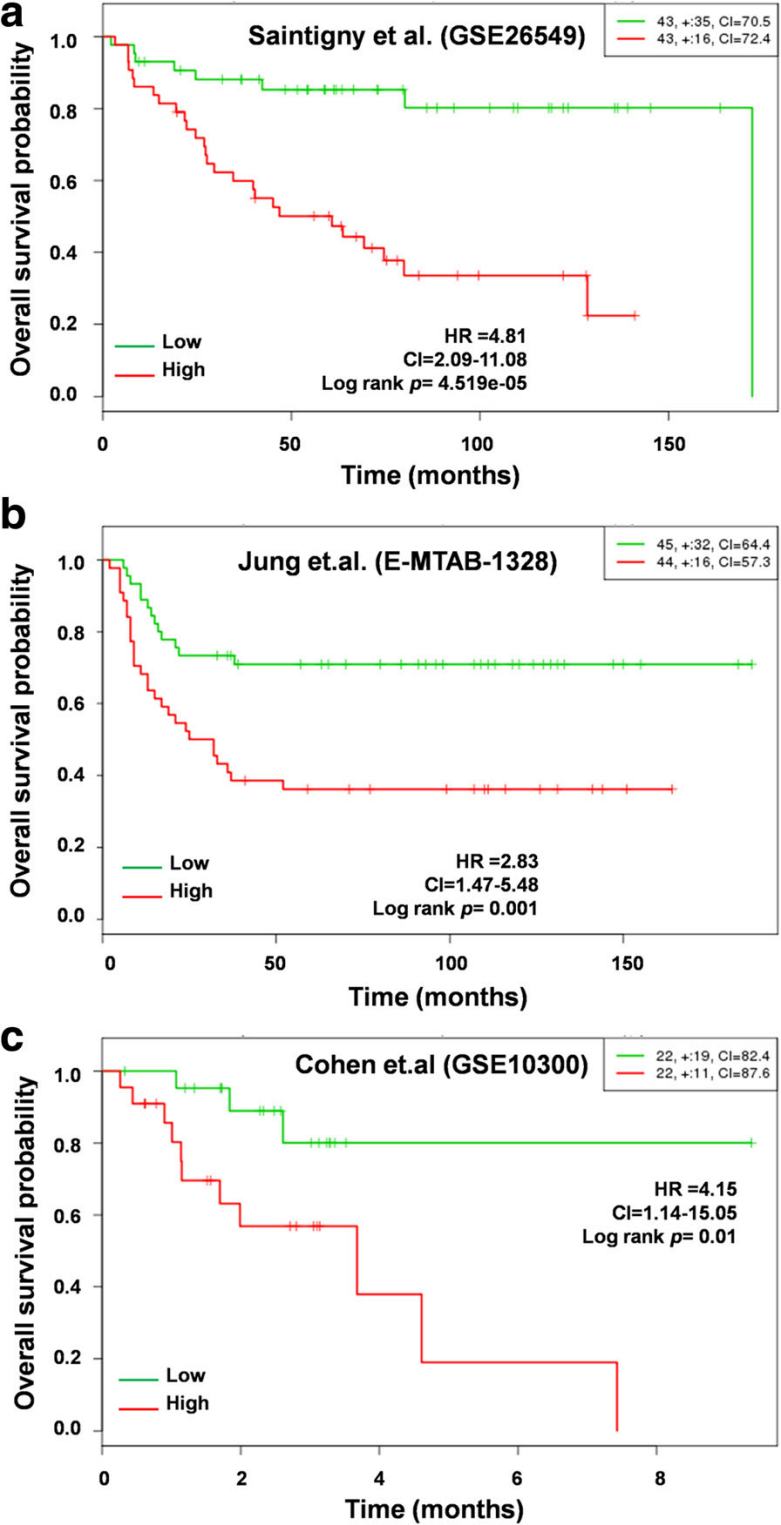
17-gene signature predicts poor survival in three independent cohorts. Kaplan-Meier survival plots showing that high expression of the 17-gene signature is associated with poor survival in 3 independent HNSCC cohorts: **a** Saintigny et al. (GSE26549). **b** Jung et al. (E-MTAB-1328). **c** Cohen et al. (GSE10300). Red, high-risk group; green, low-risk group

After analyzing the prognostic value of the 17-gene signature in the TCGA cohort, we evaluated its prognostic value in another 3 HNSCC cohorts containing DFS, MFS, and recurrence data. Among these, Saintigny et al. (GSE26549) [22] contains DFS data, while Jung et al. (E-MTAB-1328) [23] contains MFS data. The third cohort, Cohen et al. (GSE10300) [24], contains recurrence data. Interestingly, our DFS analysis using the Saintigny et al. (GSE26549) [22] cohort showed that patients in the high-risk group with increased expression of the 17-gene signature had poorer survival (HR = 2.28; CI = 1.56–3.32; *p* = 1.221e-05) than the low-risk group (Fig. 6a). Likewise, we found a markedly shorter MFS (HR = 2.83, CI = 1.47–5.48; *p* = 0.001) in the high-risk group of the Jung et al. (E-MTAB-1328) [23] cohort (Fig. 6b). In the Cohen et al. (GSE10300) [24] cohort, we found lower recurrence-free survival (HR = 4.15; CI = 1.14–15.05; *p* < 0.01) in the high-risk group with the17-gene signature than in the low-risk group (Fig. 6c). Thus, log-rank analysis revealed that the 17-gene signature was associated with a significantly increased risk of recurrence in HNSCC. The multivariate analysis results for the above cohorts were listed in Additional file 5: Table S5.

## Discussion

The TCGA network provides valuable information about genetic changes in key genes involved in the oxidative-stress pathway, such as KEAP1, NRF2, and CUL3, in HNSCC patients. These particular data permit researchers to identify potential biomarkers, druggable mutations, and therapeutic targets for personalized medicine. In this study, using a new approach that consisted of two RNA-Seq DEG analysis methods, we identified a common set of 17 genes regulated by the KEAP1-NRF2-CUL3 axis that constitute an expression signature in TCGA-HNSCC patients. We further tested this signature in 4 independent clinical cohorts including the TCGA-HNSCC cohort. Kaplan-Meier survival plots generated for all 4 cohorts showed that higher expression of this gene signature is significantly correlated with poor survival outcomes.

The DFS data of Saintigny et al. (GSE26549) [22] suggested that patients with an increased 17-gene signature had poor benefit from chemotherapy because of aggressive expression of genes downstream of NRF2 that are involved in chemoresistance. Our GO and KEGG analysis of the 17-gene signature strongly supported the above conclusion. The top two enriched GO biological process terms were ‘daunorubicin metabolic process’ and ‘doxorubicin metabolic process’, clearly indicating that the genes involved in these processes, such as AKR (aldo-keto reductase) 1C3, AKR1C2, AKR1B10, and AKR1C1, are crucial drug-metabolizing enzymes whose overexpression is strongly associated with drug resistance in many cancers [38, 39] (Table 2). Aldo-keto reductases are well-characterized NRF2-regulated genes which contain consensus ARE sequences in their promoter regions for the binding and transactivation of NRF2 [39–41]. A recent lung cancer study emphasized that a panel of aldo-keto reductase family genes are markedly upregulated in patients harboring somatic alterations in the NRF2 pathway and considered to be biomarkers of NRF2 hyperactivation in lung cancer [17]. Consistent with their study, we showed that aldo-keto reductases were not only highly expressed in lung cancer but also in HNSCC patients with a dysregulated NRF2 pathway and could be used as biomarkers.

More interestingly, the top hit in the KEGG pathway analysis of the 17-gene signature identified an important pathway involved in oxido-reductase activity known as ‘glutathione metabolism’(Table 2). The genes listed in this pathway, such as GSTM3, G6PD, GCLC, and GCLM, play major roles in redox balance in normal cells. The redox imbalance in cancer cells because of the overexpression of these genes mainly leads to tumor growth and drug resistance [42]. Thus, our study revealed that NRF2 drives the expression of genes involved in glutathione metabolism, so the development of NRF2 inhibitors could be a means of altering tumor growth and drug resistance in HNSCC. A very interesting recent study on the inhibition of NRF2, glutathione (GSH), and thioredoxin (Trx) in head and neck cancer (HNC) strongly supports our prediction that combined inhibition of the GSH, Trx, and NRF2 pathways could be an effective strategy to overcome therapeutic resistance in HNC [43].

**Fig. 7:**
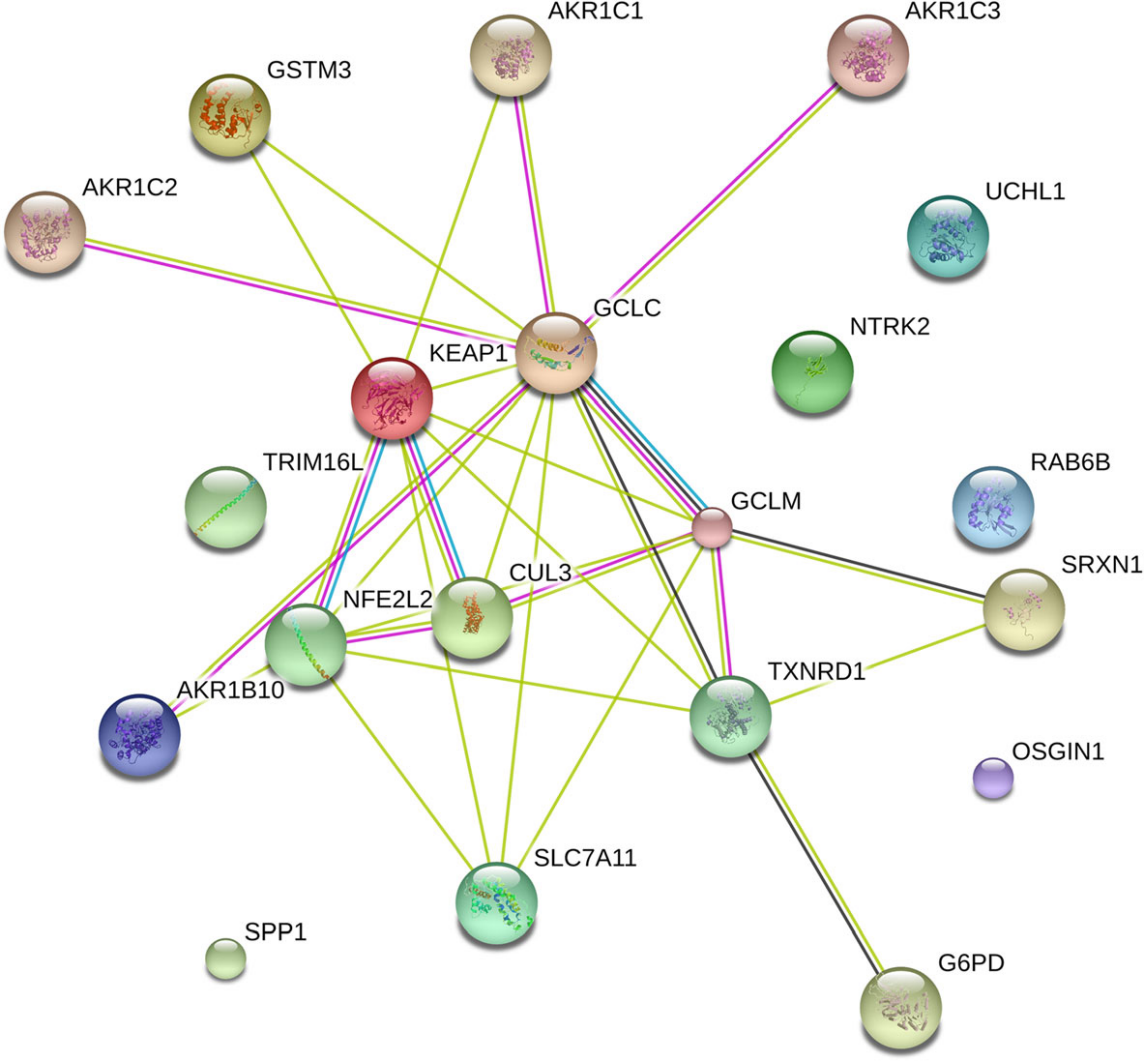
Protein-protein interaction network analysis of the 17-gene signature predicting the functional correlation of the KEAP1-NRF2-CUL3 axis with genes involved in drug metabolism and glutathione metabolic pathways in HNSCC

In addition to the GO and KEGG analyses, we used the STRING v10 database to construct a PPI network of the 17-gene signature along with the KEAP1, NRF2, and CUL3 genes to reveal the complex associations between these genes. The enrichment results based on functional association between these genes revealed that the majority were closely associated with each other through a coordinated interactive network (Fig. 7). Thus, PPI network analysis suggested that the cross-talk of KEAP1, NRF2, and CUL3 with the 17-gene signature coordinately drives tumor progression and therapeutic resistance in HNSCC.

Apart from known NRF2-regulated genes, we found 4 putative KEAP1-NRF2-CUL3 axis-regulated genes: NTRK2, RAB6B, TRIM16L, and UCHL1. NTRK2, also known as tropomyosin receptor kinase B, is a neurotrophin-binding protein that phosphorylates members of the MAPK pathway. This receptor plays a major role in cell differentiation, specifically neuronal proliferation, differentiation, and survival, through its kinase signaling cascade [44]. Emerging evidence suggests that NTRK2 plays an important role in different cancers. For instance, it has been reported to be highly expressed in non-small cell lung cancer A549 cells [45] and is associated with a worse outcome in patients with Wilms’ tumor [46].

Although NRF2-ARE sequences were not found in the NTRK2 promoter region, we looked into why NTRK2 was highly upregulated in altered samples. Surprisingly, a recent report revealed that NTRK2 inhibits KEAP1 expression in breast cancer cells and is involved in cancer proliferation, survival, and metastasis [47]. Thus, the overexpression of NTRK2 in altered samples clearly suggests that NTRK2 inhibits the expression of KEAP1, initiates the hyperactivation of genes downstream of NRF2, and is involved in HNSCC tumorigenesis. Another putative KEAP1-NRF2-CUL3 gene, UCHL1 (ubiquitin C-terminal hydrolase L1), has also been implicated in different types of human cancer such as breast [48, 49], melanoma [50], ovarian [51], colorectal [52], osteosarcoma [53], and gastric [54, 55] cancers, and multiple myeloma [56]. Most of the cancer studies on UCHL1 have revealed that overexpression and promoter methylation of UCHL1 are key reasons for UCHL1-mediated metastasis. Due to the adverse effect of overexpression of UCHL1, it is considered to be a biomarker and a therapeutic target in many cancers. The exact functions of the other two putative KEAP1-NRF2-CUL3axis-regulated genes, RAB6B and TRIM16L, are unknown in cancer cells and therefore are under investigation in our lab. Altogether, the above evidence suggests an oncogenic role of the 17-gene signature in many cancers.

## Conclusions

In conclusion, we have identified a comprehensive gene signature of the KEAP1-NRF2-CUL3 axis, increased expression of which predicts poor survival in HNSCC. Moreover, the components of this 17-gene signature can be used as potential biomarkers to identify genetic alterations of the NRF2 pathway in HNSCC. Furthermore, the development of combined inhibitors for this 17-gene signature, along with NRF2, could pave the way for the development of personalized/precision medicine to suppress NRF2-mediated tumor growth and drug resistance.

## Additional files

Additional file 1: Table S1. List of differentially expressed genes obtained from the RNA-Seq analysis of altered versus normal samples. (XLS 48 kb)

Additional file 2: Table S2. List of differentially expressed genes obtained from the RNA-Seq analysis of altered versus wild-type samples. (XLS 29 kb)

Additional file 3: Table S3. List of differentially expressed genes obtained from the RNA-Seq analysis of wild-type versus normal samples. (XLS 49 kb)

Additional file 4: Table S4. List of NRF2-AREs identified in the -5 kb promoter regions of RAB6B, UCHL1 and TRIM16L genes. (XLS 38 kb)

Additional file 5: Table S5. Multivariate analysis of 17-gene signature in 4 independent cohorts (XLS 25 kb)

Additional file 6: Figure S1. Kaplan-Meier plots showing the survival analysis of TCGA-HNSCC cohort clinical variables: tumor grades G2 (A) and G3 (B); pathologic T stagesT1 (C) and T2 (D); and pathologic disease stages II (E) and III (F). (TIFF 798 kb)

## Abbreviations

AKR1B10: Aldo-keto reductase family 1 member B10
AKR1C1: Aldo-keto reductase family 1 member C1
AKR1C2: Aldo-keto reductase family 1 member C2
AKR1C3: Aldo-keto reductase family 1 member C3
ARE: Antioxidant responsive element
BH: Benjamini-Hochberg
CUL3: Cullin-dependent E3 ligase
DAVID: Database for annotation visualization and integrated discovery
DEG: Differential Expression Genes
DFS: Disease free survival
edgeR: Empirical Analysis of Digital Gene Expression Data in R
FDR: False discovery rate
G6PD: Glucose-6-phosphate dehydrogenase
GCLC: Glutamate-cysteine ligase catalytic subunit
GCLM: Glutamate-cysteine ligase modifier subunit
GDAC: Genome Data Analysis Center
GO: Gene ontology
GSH: glutathione
GSTM3: Glutathione S-transferase mu 3
HNSCCC: Head and Neck Squamous Cell Cancer
KEAP1: Kelch like-ECH-associated protein 1
KEGG: Kyoto Encyclopedia of Genes and Genomes
MFS: Metastasis-free survival
NRF2: Nuclear factor erythroid 2-related factor
NTRK2: Neurotrophic receptor tyrosine kinase 2
OSGIN1: Oxidative stress induced growth inhibitor 1
PPI: Protein-Protein interaction
RAB6B: RAB6B, member RAS oncogene family
RBX1: Ring-Box 1
RT-qPCR: Reverse transcription–quantitative polymerase chain reaction
SLC7A11: Solute carrier family 7 member 11
SPP1: Secreted phosphoprotein 1
SRXN1: Sulforedoxin 1
TCGA: The Cancer Genome Atlas
TRIM16L: Tripartite motif containing 16-like
TrkB: Tropomyosin receptor kinase B
Trx: Thioredoxin
TXNRD1: Thioredoxin reductase 1
UCHL1: Ubiquitin C-terminal hydrolase L1
