# Supplementary figures and images for: Gene-expression signature regulated by the KEAP1-NRF2-CUL3 axis is associated with a poor prognosis in head and neck squamous cell cancer
Source: BMC Cancer. 2018 Jan 6;18:46. doi: 10.1186/s12885-017-3907-z (PMC5756380; doi:10.1186/s12885-017-3907-z)

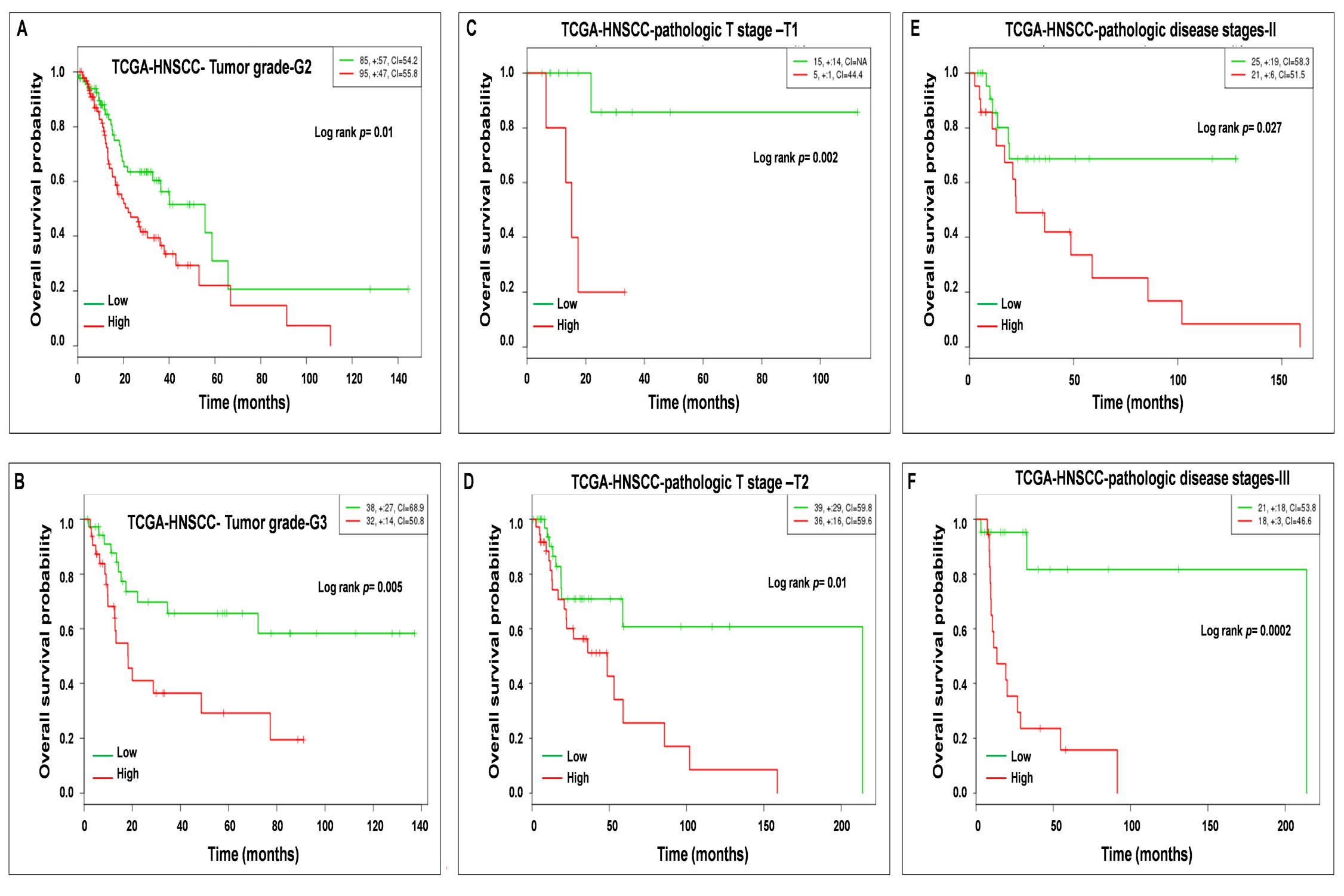

Supplement: Supplementary file 6 — Kaplan-Meier plots showing the survival analysis of TCGA-HNSCC cohort clinical variables: tumor grades G2 (A) and G3 (B); pathologic T stagesT1 (C) and T2 (D); and pathologic disease stages II (E) and III (F). (TIFF 798 kb) [file 12885_2017_3907_MOESM6_ESM.tif]
